# Evaluating the performance of diagnostic methods for soil transmitted helminths in the Amhara National Regional State, Northwest Ethiopia

**DOI:** 10.1186/s12879-020-05533-2

**Published:** 2020-10-29

**Authors:** Abebe Fenta, Tadesse Hailu, Megbaru Alemu, Endalkachew Nibret, Arancha Amor, Abaineh Munshea

**Affiliations:** 1 Health Science College, Debremarkos University, P.o.box: 269, Debre Markos, Ethiopia; 2 College of Medicine and Health Sciences, Bahir Dar University, P.O.Box: 79, Bahir Dar, Ethiopia; 3 Science College, Bahir Dar University, P.O.Box: 79, Bahir Dar, Ethiopia; 4 Mundo Sano Foundations, Institute of Health Carlos III, Madrid, Spain

**Keywords:** Soil-transmitted helminths, Diagnostic performance, Method comparison, School children, Ethiopia

## Abstract

**Background:**

Soil-transmitted helminths are more prevalent in tropics and sub-tropics including Ethiopia. Despite their high prevalence, direct saline microscopy with its low sensitivity has been used as a diagnostic method in almost all health facilities in Ethiopia. Alternative diagnostic methods which have higher sensitivity are not yet implemented. Therefore, this study aimed to compare and evaluate the performance of diagnostic methods for soil transmitted helminths.

**Methods:**

A cross-sectional study among 520 school children was conducted from October to December, 2019 in Amhara National Regional State. The study participants were selected using systematic random sampling technique. Stool samples were processed via formol ether concentration, Kato-Katz, spontaneous tube sedimentation and agar plate culture techniques. Data was entered into Epi-data version 3.1 and analysis was done using SPSS version 20.0. The sensitivity, specificity, and negative predictive value were calculated against the combined result. Strength of agreement of the diagnostic methods was determined by Kappa value.

**Results:**

The Overall prevalence of soil transmitted helminths was 40.8% using combination of methods. The prevalence 24.4, 22.5, and 32.4%, respectively was recorded by using formol ether concentration, Kato-Katz and spontaneous tube sedimentation. The highest prevalence of hookworm (29.2%) was detected by the agar plate culture. The sensitivity and negative predictive value of formol ether concentration were 57.9 and 78.4%, for Kato-Katz thick smear 55.2 and 76.4%, for spontaneous tube sedimentation were 79.2 and 87.5% to soil transmitted helminths detection, respectively. The sensitivity and negative predictive value of agar plate culture to hookworm detection were 86.4 and 93.5%, respectively.

**Conclusion:**

Spontaneous tube sedimentation shows higher sensitivity in the detection of soil transmitted helminth infections. Agar plate culture method also indicated better performance for hookworm detection than other methods. Therefore, the employment of spontaneous tube sedimentation technique for routine laboratory and agar plate culture for research purposes will significantly aid in accurate diagnosis of parasitic infections.

## Background

The soil-transmitted helminths (STHs) that infect human include, *Ascaris lumbricoides*, *Trichuris trichiura* and hookworm species (*Necator americanus* and *Ancylostoma duodenale*) [1]. The global burden of disease due to STHs infections is estimated at 5.2 million disability-adjusted life years (DALYs) [2]. In Ethiopia, the number of people living in STHs endemic areas are estimated at 79 million [3, 4]. The regional prevalence within the Amhara region was 36.4% for any STHs [5].

Transmission of STHs is through ingestion of eggs with food, water, soil and/or contaminated fingers or percutaneously by larvae depending on the species [6]. Once STHs enter the human system, they chronically infect children and result in chronic malnutrition and anaemia leading to physical and mental abnormality [7].

Several diagnostic techniques including; direct wet mount, Kato-Katz (KK), Spontaneous tube sedimentation (STS), Formol-ether concentration (FEC), agar plate culture (APC), immunodiagnostic and molecular techniques are employed however, they vary in their sensitivity, cost, simplicity and applicability [8, 9]. The KK technique is mainly recommended to detect STHs infections [10, 11]**.** In effective diagnosis, detection methods must be accurate, simple and affordable for the whole population and provide results in a short period of time to effective prevention and control measures [12].

Low sensitive diagnostic methods like the wet mount technique leads to under diagnosis of STHs infections and may mislead physicians [13–16]. Proper detection of STHs using sensitive diagnostic methods is crucial in the national STHs prevention and control strategies. However, direct wet mount is still used as a routine diagnostic method for STHs infections in Ethiopia with its low sensitivity. This has a negative impact on the prevention and control plan. To push the STHs prevention and control strategies one step forward, updating and applying better sensitive, specific and cost effective diagnostic methods as a routine diagnostic approach is ideal in Ethiopia. This might reduce under diagnosis and under report of STHs infections in the country. Therefore, the aim of this study was to evaluate the performance of FEC, KK, STS and APC in STHs detection in Amhara National Regional State (ANRS).

## Method

### Study design, period and area

A cross-sectional study was conducted among primary school children from October to December, 2019 in ANRS. The ANRS consists of 10 zones and 157 districts and is divided into three major ecological zones: the highlands, midlands and the lowlands. The annual mean temperature is between 15 °C and 21 °C. The mean annual rainfall is also 1165 mm. According to the economic and finance office of the ANRS 2017 report, the total population of children (5–14 years) in the region is 5,996,074 [17].

Six districts and twelve primary schools (two primary schools in each district) were selected by simple random sampling technique and a systematic random sampling technique was used to select the study participants in each selected primary school as class roster was used as a sampling frame. The sample size was proportionally allocated for each school by taking the total number of students in each category into consideration.

Students who fall in the age range 6–14 years, gave consent and volunteered to participate were included whereas, students who took anti-helminthic drugs for the last 2 months prior to data collection or during data collection time were excluded from the study.

### Data collection and processing

Approximately, 10 g of fresh stool sample was collected with 25.0 ml stool cups from each study participant and transported to the nearby health institution within an hour. The fresh stool samples were processed with FEC, KK, STS and APC methods to detect STHs.

In the modified Richie’s method, approximately 0.5 g fresh stool sample was added in the sample collecting tube containing 2.5 ml of formalin and 1 ml of ethyl acetate. The test tube was mixed well and centrifuged at 1500 revolutions for three minutes. Finally, the supernatant was discarded and the sediment was mixed and put on the microscope to detect the ova and the larva of STHS [18].

In the KK technique, a stool sample was pressed through a mesh screen to remove large particles. About 41.7 mg of sieved stool was transferred to the templates which was put on a slide till the template whole is filled. Then, the template was removed and the stool sample was covered and pressed with cellophane which is previously immersed with Glycerol-malachite green. The KK thick smears were examined within 30 min for hookworm and after one hour up to 24 h for other intestinal parasites [11].

In the STS technique, approximately 3 g of fresh stool sample was weighed and homogenized in 10 ml of normal saline solution. The mixture was filtered through surgical gauze into a 50 ml plastic tube which was then filled with more saline solution to 50 ml gauge, plugged, and shaken vigorously. The tube was left to stand for 45 min, and then discard the supernatant. A sample was taken from the bottom and put on a microscope slide and seen for ova and larva of STHs [9].

In APC technique, about 3 g of fresh stool was placed on the centre of a primary APC in a 100 × 15 mm Petri dish. The Petri dish was sealed with adhesive tape and incubated at26 °C) for two days. The surface of the agar-plate was analyzed daily with an inverted microscope for the presence of tracks of moving larvae. Then after, 5 ml of a 10% formalin solution was added to the agar surface and waited for 5 min. The excess formalin was collected with a conical test tube and centrifuged at 1500 for 5 min. Finally, the sediment was first seen for larvae of hookworm with microscope [19].

### Performance evaluation

The detection rate and performance of FEC, STS, KK and APC methods to STHs was checked. The diagnostic agreements of methods were evaluated by Kappa value, number of observed agreements, number of agreements expected by chance and standard error of Kappa. Kappa result was interpreted as follows: values ≤0 as indicating no agreement and 0.01–0.20 as none to slight, 0.21–0.40 as fair, 0.41–0.60 as moderate, 0.61–0.80 as substantial, and 0.81–1.00 as almost perfect agreement [20].

### Data quality control

Training was given for laboratory personnel about the study, data collection, and detection. The quality of reagents and instruments were checked. The stool samples were also checked for serial number, and quantity. To eliminate observer bias, each stool sample was examined immediately by two laboratory personnel. To maintain the reliability of the study findings, 15% of the results of each method were randomly selected and re-examined by a third laboratory personnel who was blind for the first stool examination. The principal investigator checked the discordant results and put the final result.

### Data analysis

Data was entered in Epi-data version 3.1 and analyzed using SPSS version 20.0 statistical software for descriptive statistics. The sensitivity, specificity, negative predictive value and positive predictive values for each diagnostic method in STHs detection was calculated against the combined results as a “Gold” standard method. Kappa values were estimated at 95% CI to determine the strength of agreement of the diagnostic methods. *P*-value < 0.05 were considered as statistically significant.

### Ethical consideration

Ethical clearance was obtained from College of Medicine and Health Science ethical review committee, Bahir Dar University and permission letter was obtained from ANR health bureau. Supporting letters were also obtained from ANR education bureau, Zonal and district education offices. Written informed consent was secured from parents/guardians of each study participant. Study participants infected with intestinal parasites were referred to doctors at the nearby health institution.

## Results

### Socio-demographic characteristics of the study participants

A total of five hundred twenty (*n* = 520) students were enrolled in this study. The mean age of study participants was 10.14 years ranged from 6 to 14 years with a standard deviation of 1.66 years. The male participants accounted for 266 (51.2%). Four hundred ninety seven (95.6%) participants were rural dwellers and 459 (88.3%) of the participants were Orthodox in their religion.

### Prevalence of STHs

From the total enrolled study subjects, the prevalence of parasites identified were 7.7, 33.8, 0.8, 20.2, 1.3, 8.1, 3.3, 16.5 and 8.3% with the respective *A. lumbricoides*, hookworm, *T. trichiura, S. mansoni*, *E. vermicularis, S. stercoralis*, *H. nana*, *E. histolytica/dispar* and *G. lamblia* with a combined method. The overall prevalence of STHs was 40.8% with a combined method. The prevalence of hookworm, *A. lumbricoides* and *T. trichiura* infections by combined methods was 33.8, 7.7 and 0.8%, respectively (Table 1). The detection rate 32.4, 24.4 and 22.5% to STHs infections was obtained using STS, FEC, and KK techniques, respectively. The prevalence of hookworm infection via the APC technique was 29.2% (Table 1).

**Table 1:** The prevalence of STHs using Combined, FEC, KK, STS and APC techniques among school children in ANRS, from October to December,2019.(*n* = 520)

Parasite	Diagnostic technique	Prevalence detected by each method		
		N	%	95% CI
*A. lumbricoides*	Combined	40	7.7	5.4–10
	FEC	13	2.5	1.2–3.8
	KK	20	3.8	2.2–5.5
	STS	32	6.2	4.2–8.4
Hookworm	Combined	176	33.8	29.8–37.9
	FEC	113	21.7	18.2–25.3
	KK	98	18.8	15.5–22.2
	STS	137	26.3	22.5–30.1
	APC	152	29.2	25.3–33.2
*T. trichiura*	Combined	4	0.8	0.02–1.5
	FEC	3	0.6	0.08–1.2
	KK	3	0.6	0.08–1.2
	STS	4	0.8	0.02–1.5
STHs	Combined	212	40.8	36.6–45.1
	FEC	127	24.4	20.1–28.3
	KK	117	22.5	19.1–26.3
	STS	168	32.4	28.4–36.5

*N* number positive, *CI* Confidence interval

### Detection and performance evaluation of diagnostic methods for STHs

The detection rate of the combined methods to STHs was 1.81, 1.67, and 1.26 times more sensitive than KK, FEC, and STS, respectively. The detection rate of the STS method to STHs was also 1.44 and 1.32 times more sensitive than KK and FEC methods, respectively (Table 2).

**Table 2:** The sensitivity, specificity, NPV and PPV of FEC, KK, STS and APC techniques for the diagnosis of STHs against the gold standard among school children in ANRS, from October to December, 2019. (*n* = 520)

			“Gold” standard					
Method	Species	Result	Pos	Neg	Sensitivity	Specificity	NPV	PPV
					% (95% CI)	% (95% CI)	% (95% CI)	% (95% CI)
FEC	AL	pos	13	0	32.5 (20.1–48.0)	100 (99.2–100)	94.7 (92.4–96.3)	100 (77.2–100)
		Neg	27	480				
	HW	pos	113	0	64.2 (56.9–70.9)	100 (98.9–100)	84.5 (80.7–87.7)	100 (96.7–100)
		Neg	63	344				
	TT	pos	3	0	75.0 (31.0–95.4)	100 (99.3–100)	99.8 (98.9–100)	100 (43.9–100)
		Neg	1	516				
	STHs	pos	127	0	57.9 (51.0–64.5)	100 (98.8–100)	78.4 (74.0--82.2)	100 (96.8–100)
		Neg	85	308				
KK	AL	pos	20	0	50.0 (35.2–64.8)	100 (99.2–100)	96.0 (93.9–97.4)	100 (83.9–100)
		Neg	20	480				
	HW	pos	98	0	55.7 (48.3–62.8	100 (98.9–100)	81.5 (77.5–84.9)	100 (96.2–100)
		Neg	78	344				
	TT	pos	3	0	75.0 (31.0–95.4)	100 (99.3–100)	99.8 (98.9–100)	100 (43.9–100)
		Neg	1	516				
	STHs	pos	117	0	55.2 (48.5–61.7)	100 (98.8–100)	76.4 (72.0–80.3)	100 (96.8–100)
		Neg	95	308				
STS	AL	pos	32	0	80.0 (65.2–89.5)	100 (99.2–100)	98.4 (96.8–99.2)	100 (89.3–100)
		Neg	8	480				
	HW	pos	137	0	77.8 (71.1–83.3)	100 (98.9–100)	89.8 (86.4–92.5)	100 (97.3–100)
		Neg	39	344				
	TT	pos	4	0	100 (51.0–100)	100 (99.3–100)	100 (99.3–100)	100 (51.0–100)
		Neg	0	516				
	STHs	pos	168	0	79.2 (73.3–84.2)	100 (98.8–100)	87.5 (83.6–90.6)	100 (97.8–100)
		Neg	44	308				
APC	HW	pos	152	0	86.4 (80.5–90.7)	100 (98.9–100)	93.5 (90.5–95.6)	100 (97.5–100)
		Neg	24	344				

*AL A. lumbricoides*, *HW* Hookworm, *TT T. trichiura*, *CI* confidence interval, *PPV* positive predictive value, *NPV* negative predictive value

Better detection rate when two techniques used at a time was 35.8% by STS and KK, followed by 35.4% by STS and FEC, and 29.2% by KK and FEC in the identification of STHs infection. A combination of three methods (FEC, STS and KK) had higher detection rate (37.5%) than combination of two methods (Table 3).

**Table 3:** The detection rate of FEC, KK and STS techniques individually and their combinations for the diagnosis of STHs parasites among school children in ANRS, from October to December, 2019. (*n* = 520)

Methods	Total examined(N)	STHs	
		Pos (N (%))	% (95% CI)
FEC	520	127 (24.4)	20.1–28.3
KK	520	117 (22.5)	19.1–26.3
STS	520	168 (32.4)	28.4–36.5
FEC + KK	520	152 (29.2)	25.5–33.3
FEC + STS	520	184 (35.4)	31.4–39.6
KK + STS	520	186 (35.8)	31.8–40
FEC + KK + STS	520	195 (37.5)	33.4–41.7

*Pos* positive, *Neg* negative, *N* number

The STS method (79.2%) had better sensitivity as compared to KK (55.2%) and FEC (57.9%) methods in STHs detection (Table 2). The sensitivity of KK technique for *A. lumbricoides* (50%) infections was higher than FEC technique sensitivity for *A. lumbricoides* (32.5%) infection (Table 2). The APC technique had better sensitivity (86.4%) and NPV (93.5%) in diagnosing hookworm infection compared to the STS (Sensitivity (77.8%) and NPV (89.8%)), FEC (Sensitivity (64.2%) and NPV (84.5%)) and KK (Sensitivity (55.7%) and NPV (81.5%)) techniques. However, all the four techniques had 100% specificity and positive predictive values in STHs detection (Table 2).

### Agreement of the diagnostic methods

The observed and expected agreements between STS method and combined method were 91.54 and 53.27% of the observations, respectively for the identification of STHs infection. The respective observed and expected agreements 95.38 and 56.71% were obtained between APC method and combined method in the detection of hookworm infection (Table 4). The agreement of STS technique with the combined results was perfect in detecting STHs (*к*=0.819) (Table 4). The KK method agreed perfectly in *T. trichiura* (*к*=0.856), substantially in *A. lumbricoides* (*к*=0.649) and hookworm (*к*=0.624) detections with combined techniques (Table 4). The FEC technique agreed perfectly in *T. trichiura* (*к*=0.856), substantially in hookworm (*к*=0.704), and moderately in *A. lumbricoides* (*к*=0.471) detections with gold standard (Table 4).

**Table 4:** Test agreement of FEC, KK, STS and APC techniques to detect STHs and *S. mansoni* against the gold standard among school children in ANRS, from October to December, 2019. (*n* = 520)

	Combined as a “Gold” standard							
Methods	Species	Result	Pos (N)	Neg (N)	NOA	NAEC	Kappa-value; (*p*-value)	95% CI of kappa
					N (%)	N (%)		
FEC	AL	Pos	13	0	493 (94.81)	469 (90.19)	0.471 (0.001)	0.306–0.636
		Neg	27	480				
	HW	Pos	113	0	457 (87.88)	307 (59.5)	0.704 (0.001)	0.638–0.769
		Neg	63	344				
	TT	Pos	3	0	519 (99.81)	513 (98.66)	0.856 (0.001)	0.578–1.00
		Neg	1	516				
	STHs	Pos	127	0	435 (83.65)	284.6 (54.72)	0.639 (0.001)	0.574–0.704
		Neg	85	308				
KK	AL	Pos	20	0	500 (96.15)	463.1 (89.05)	0.649 (0.001)	0.507–0.790
		Neg	20	480				
	HW	Pos	98	0	442 (85.00)	312.3 (60.07)	0.624 (0.001)	0.553–0.696
		Neg	78	344				
	TT	Pos	3	0	519 (99.81)	513 (98.66)	0.856 (0.001)	0.578–1.00
		Neg	1	516				
	STHs	Pos	117	0	425 (81.73)	286.4 (55.08)	0.593 (0.001)	0.526–0.661
		Neg	95	308				
STS	AL	Pos	32	0	512 (98.46)	452.9 (87.10)	0.881 (0.001)	0.799–0.962
		Neg	8	480				
	HW	Pos	137	0	481 (92.50)	299.7 (57.64)	0.823 (0.001)	0.770–0.876)
		Neg	39	344				
	TT	Pos	4	0	520 (100)	512 (98.47)	1.00 (0.001)	1.00–1.00
		Neg	0	516				
	STHs	Pos	168	0	476 (91.54)	277 (53.27)	0.819 (0.001)	0.769–0.869
		Neg	44	308				
APC	HW	Pos	152	0	496 (95.38)	294.9 (56.71)	0.893 (0.001)	0.852–0.935
		Neg	24	344				

Note: *AL A. lumbricoides*, *HW* Hookworm, *TT T. trichiura*, *NOA* Number of observed agreements, *NAEC* Number of agreements expected by chance, *CI* confidence interval

Furthermore, the *к* agreement between the APC and gold standard for the diagnosis of hookworm was perfect (*к*=0.893) (Table 4).

## Discussion

Accurate and sensitive diagnostic methods are necessary for the clinical and public health diagnosis of helminth infections, as well as for monitoring treatment and control interventions [21]. The rate of STHs in the present study was 40.8% which goes in line with previous findings in the Amhara region (36.4%) [5]. The present study is also lower than the previous result recorded 52.4% in lake Hawassa [22], but this result is higher than the prevalence obtained 25.8% in Peruvian Amazon [23]; 5.5% in Zimbabwe [24]; 6.73% in Tanzania [25]; 16.2% in Kenya [26]; 14.5% in Jawi [27] and 36.2% in Bahir Dar [28]. These differences could be owing to differences in geographical, ecological, season of data collection and different diagnostics.

The STS technique is the simplest, fastest method to perform, requires less equipment and detects many species [9]. The detection rate of the STS method was 1.32, 2.48, and 1.33 times more sensitive than the FEC method to detect STHs, *A. lumbricoides* and *T. trichiura,* respectively. This result agrees with previous cross sectional study conducted in Peru [9]. The detection rate of STS method in the diagnosis of *A. lumbricoides*, hookworm and *T. trichiura* were also 1.63, 1.4 and 1.33 times more sensitive than KK method, respectively which is consistent with a study conducted in Peru by both methods [29].

In the present study, STHs were found in 24.4% of the students, using FEC technique and 22.5% by KK method. The difference in their diagnostic sensitivity is statistically significant (*P* = 0.001) which showed that FEC method was more sensitive than KK method. This result agrees with the study done in Ethiopia [30, 31], Coˆ te d’Ivoire [32] and Tanzania [21]. However, the result of the present study is in contrast to previous studies done in Peusangan Bireuen [33] and Ethiopia [34, 35]. These differences in diagnostic performance might be due to stool sample size, number of slides prepared for diagnosis, interpersonal skill variations and technical errors of the two methods.

The detection rate of FEC method in the identification of hookworm infection was 21.7% which goes in line with findings from Bahir Dar City (20.6%) [30] and rural Bahir Dar (23.7%) [36]. The FEC method was 1.2 times more sensitive in hookworm detection than KK method in this study. Consistence results of FEC detection rate obtained 1.1 and 1.03 times more sensitive than KK method in Bahir Dar city and Gondar town respectively are reported previously [30, 34]. The possible justification might be due to hookworm egg disintegrated quickly due to glycerine if not observed within an hour [11, 37, 38], rapid degeneration of delicate hookworm eggs with time [16] and small amount of stool samples was processed in KK technique [29].

In the current study, the detection rate of APC method was 29.2% for the diagnosis of hookworm infection. Lower detection rate findings are recorded (13.6%) in Colombia [39], (10.7%) in Thailand [40] and (7.9%) in Tanzania [41]. These differences in diagnostic performance might be due to areas endemicity, stool sample size, interpersonal skill variations and technical errors of the diagnostic method. The detection rate of APC method to hookworm infection was 1.55 times more sensitive than KK method. Similar findings were also reported in Colombia [39] and Tanzania [41]. The APC method (29.2%) had also a higher detection rate than the FEC method (21.7%) for the diagnosis of hookworm, as reported in other previous studies [32, 42]. The APC method was also more sensitive than the STS method for the diagnosis of hookworm infection as previously described by another author in Brazil [43].

In the present study, by taking the combined results as a “Gold” standard, the sensitivity of STS and KK techniques were 79.2 and 55.2%, respectively in STHs detection. This result was quite different from previous study done in Peru reported that each KK and STS technique had 100% sensitivity for the detection of STHs [29]. The difference in diagnostic performance might be due to variations in interpersonal skill, small sample size and the “Gold” standard method used which is only a combination of KK and STS in the previous study. In the present study, the STS technique (79.2%) was more sensitive than FEC technique (57.9%) for the diagnosis of STHs infection. However, lack of previous similar studies made difficulty in making rigorous discussion on this finding.

In the present study, the FEC method and KK method had 57.9 and 55.2% sensitivity for STHs detection, respectively. The detection difference was statistically significant (*p* = 0.001). This result was consistent with similar studies done previously that showed that the FEC method has a lower sensitivity than KK method for the detection of STHs with the exception of hookworm infections [33–35]. However, the result of the present study is in contrary to the study done in Coˆ te d’Ivoire and Tanzania which showed that FEC method was more sensitive than KK method [21, 32]. These differences in diagnostic performance might be due to interpersonal skill variations and technical errors of the two methods. There might also be differences in gold standards and sample size used.

The sensitivity of APC method for hookworm detection was 86.4% which is comparable with previous study done in Colombia (76–92%) [39]. This finding is also higher than previous study results (77.6%) in Brazil [43], (45.2%) in Coˆ te d’Ivoire [32] and (49%) in Tanzania [41], but lower than previous finding (100%) in Thailand [40]. The difference could be occurring due to inter personal variations, different “Gold” standards, technical errors and also quality of the media.

The agreement of STS technique with the combined results was perfect in detecting STHs species (*A. lumbricoides* *к*=0.881, hookworm *к*=0.823 and *T. trichiura* *к*=1.00). There is no earlier data which have been conducted on the agreement of STS method with “Gold” standard method. But, the agreement result obtained in our study supports STS can be used as an alternative diagnostic method for STHs infections. This result also indicates that there is a need to make evaluation of the agreements of STS techniques rigorously with the “Gold” standard before using as a routine diagnostic method. The FEC technique agreed perfectly in *T. trichiura* (*к*=0.856), substantially in hookworm (*к*=0.704) and moderately in *A. lumbricoides* (*к*=0.471) detections with “Gold” standard. on the other hand, the agreement between the FEC method and single KK method was substantial for *A. lumbricoides* (κ = 0.62), moderate for *T. trichiura* (κ = 0.53) and fair for hookworm (κ = 0.34) in previous study done in Tanzania [21]. The difference could be arising due to the “Gold” standard method.

## Conclusions

The present study revealed that STS method was more sensitive as compared to FEC and KK methods to detect STHs. Moreover, the APC method showed better performance for hookworm detection than the other three methods. Therefore, the employment of STS technique as a confirmatory test in routine laboratory and APC for research purposes will significantly aid in accurate diagnosis of parasitic infections in school children.

## Data Availability

The original data for this study is available from the corresponding author. Therefore, minimal data could be accessed upon request.

## Abbreviations

ANRS: Amhara National Regional State
APC: Agar plate culture
DALYs: Disability-adjusted life Years
FEC: Formal-ether concentration
KAP: Koga agar plate
KK: Kato Katz
MDA: Multi-drug administration
NPV: Negative predictive value
PPV: Positive predictive value
SSA: Sub-Saharan Africa
STHs: Soil transmitted helminths
STS: Spontaneous tube sedimentation
WHO: World Health Organization
